# 

**Published:** 1834-01-01

**Authors:** 


### METEOROLOGICAL REGISTER,

### NOTICES.

*We have received* ADAMS' *translation of Paulus Ægineta*, COLQUHOUN *on Animal Magnetism*, Sir C. SCUDAMORE *on Inhalation, and* WALKER *on the Physiology of the Iris. We shall review them in our next.*

*We have also received* Dr. EPPS'S *Essay on Counteraction.*

*Advertisements of books about to be published can appear only in our Advertising Sheet.*

*We are much obliged to* Dr. PEACOCK *for his communication: he will find an abstract of it in our Collectanea.*

*We shall be happy to hear again from* Mr. VALENTINE.

### INDEX TO VOL. I.

PAGE.
ABDOMEN, wounds of the, case of . . . 240
Abdominal abscess after parturition, case of, by Mr. Valentine . 425
Aberdeen and Edinburgh, their fertility in degrees . . 223
Abortion, remarkable case of . . . . . 190
Abracadabra, meaning of the term . . . . 132
Abscess near the rectum, case of . . . . 114
Acetate of lead, its doses . . . . . 119
Aldersgate-street Dispensary . . . . . 226
Alibert on Diseases of the Skin, reviewed . . 144 *et seq.*
Amaurosis, cases of . . . . 26, 430, 431
Ammonia, good and bad methods of prescribing . . 234
Anastomosing aneurism of the external maxillary artery, cured by tying the common carotid . . . . . 417
Anatomical plates, by Dr. Quain, reviewed . . 374
Anatomy Act; its operation . . . . 450
the ancients not ignorant of . . . . 6
Aneurism, case of supposed . . . . . 233
Animal heat . . . . . . 255
Apoplexy, case of . . . . . 442
Arboreous ferns . . . . . . 460
Arnott, Mr.; case in which he performed œsophagotomy . . 290
Arrow-root; its microscopical characters . . . 217
Arterial laceration, case of . . . . . 170
Ascites, remarkable case of . . . . . 427
consequent on amenorrhœa; its treatment . . 444
Auscultation. A Rational Exposition of the Physical Signs of the Diseases of the Lungs and Pleura, &c., by Dr. Williams, reviewed . . 372
Authors, longevity of . . . . . . 219
Ayre, Dr., on Cholera, reviewed . . . 68 *et seq.*
his method of treating cholera . . 68, 69
Baths, medicated . . . . . . 413
Beer; his great merit . . . . . . 1
Bell, Sir Charles, on the Theory of Vision, quoted . . 250
The Hand, its Mechanism, &c., reviewed . 358
Belladonna as a prophylactic agent against scarlet fever . 186
Bills of mortality for the year 1833 . . . . 463
Benelli's solution . . . . . . 438
Boerhaave; his method of curing epilepsy . . . 92
Borelli on the walk of quadrupeds . . . . 195
Bostock, Dr., on the Theory of Vision, quoted . . 249
Bougie too often used in slight cases of permanent stricture . 163
Brain, softening of, case of . . . . . 166
function of the . . . . . 252
Brewster, Sir D., on the Theory of Vision, quoted . . 249
Bright, Dr., on Disease of the Pancreas and Duodenum . 279
Brodie, Mr., on Diseases of the Joints, quoted . . . 233
on Diseases of the Urinary Organs, reviewed . 297 *et seq.*
Bronchocele, Beck on, reviewed . . . 17 *et seq.*
can generally be cured by the knife . . 17
cases of, cured by operation . . . 20
fatal cases of . . . . . 24
Mr. Bramley's account of this disease in Nipâl . 327
Brown, Dr. Thomas; his observations on the muscular sense . 248
Burnett, Mr. his Outlines of Botany, reviewed . 93 *et seq.*
Bushnan, Mr. John Stevenson; his translation of Dieffenbach on the Restoration of the Nose, reviewed . . . 264 *et seq.*
History of a Case in which Animals were found in Blood drawn from the Veins of a Boy, reviewed 364 *et seq.*
Calculus not uncommon in India . . . . 330
Campbell's, Dr., Introduction to Midwifery, reviewed . 348 *et seq.*
Cancer of the Rectum, cases of . . . . 116

Carus on dissecting and preparing animals for collections . . 207

Castle, Mr. his Synopsis of Systematic Botany, reviewed 147 *et seq.*

Castor-oil, idiosyncrasy unfavourable to the administration of . 118

Cat, anecdote of a . . . . . 458

Celsus quoted . . . . . . 6

Chemical Diagrams, &c., by Alexander Lee, reviewed . . 369

Children should be warmly clothed . . . . 191

Cholera; its pathology . . . . 73

Dr. Ayre's cases of . . . 78 *et seq.*

cured by sulphate of quinine . . . 186

table of cases of, at Aix-la-Chapelle . . . 187

on board a French frigate . . . 190

cured by Mr. Steart with Liquor Ammoniæ . . 327

The Origin and Progress of the Malignant Cholera at Manchester, by Dr. Gaulter, reviewed . . 341 *et seq.*

treated with tartar emetic at Manchester . . 347

patients, post-mortem convulsions in . . . 432

treated by Dr. Peacock with calomel alone . . 440

Climacteric decay, cases of . . . . ib.

College of Physicians . . . . 221

College of Surgeons . . . . . 224

Colon, stricture of the, remarkable case of . . . 149

Commercial travellers; their employment in itself healthy . 208

Congestion in the liver causes cholera . . . 74

Constipation from want of fæces . . . . 115

, case of, from softening of the spinal cord . ib.

Convulsions accompanying the second dentition . . . 445

Cooper, Mr. B. B.; his case of arterial laceration . . 170

Surgical Essays, reviewed . 229 *et seq.*

Corrosive sublimate incompatible with Inf. Anthen. . . 235

Cost of becoming a man of letters . . . . 459

Couching, Mr. Raleigh on . . . . 324

Crabs' eyes, powder of, occasioned symptoms of arsenical poisoning 118

Cretins of Salzburg, account of the . . . 441

Croton oil, strength of different varieties of . . . 330

Cubebs, enema of . . . . . 120

Cyclopædia of Practical Medicine, edited by Drs. Forbes, Tweedie, and Conolly, reviewed . . . . 366 *et seq.*

Cystocele occurring during labour . . . 87

Darwall, Dr.; his death . . . . . 228

Date palm, account of the . . . . 452

Davies, Dr. Henry; Cases extracted from his Note-book . 157, 376

Davies, the tea-dealer, pronounced mad for buying an estate . 27

Davis, Dr. D., on Midwifery, reviewed . . 82 *et seq.*

Delirium, J. Hunter's Observations on . . . 313

Dengue, a variety of rheumatic fever . . . 134

Diabetes, case of . . . . . 279

Dictionary of Practical Medicine, by Dr. Copland, reviewed . 372

Dictionnaire Universel de Matière Médicale, &c., reviewed . 153

Dieffenbach's operation for the restoration of the nose . . 268

Difficulties overcome by science . . . . 458

Dislocation of the humerus upon the dorsum of the scapula . . 237

Dislocations of the patella, Mr. B. Cooper's account of . 239

Mr. Mayo's account of . . 240

Dispensaries . . . . . 467

self-supporting; their great advantages . . 468

Drying water, art of, taught by Professor Rennie . . 258

Dunglison, Dr., his Dictionary of Medical Science and Literature, reviewed 132

Earle, Mr. William, on the Functions of the Nerves, reviewed . 124 *et seq.*

divides the nerves into four classes . . 124

Edinburgh Medical and Surgical Journal, No. CXVII., reviewed 335 *et seq.*

Effusion in the brain, common in children after chronic disease . 354

Elliotson, Dr., on the discharge of fatty matters from the bowels . 287

Empyema, case of . . . . . 174

Empyema; its symptoms . . . . . 180
diagnosis of . . . . . 184
treatment of . . . . . 185
case of, successfully treated by Dr. Roe . . 423
English eye-surgery . . . . . 454
Equivocal generation . . . . . . 365
Erysipelas of the face, case of . . . . . 435
Esquirol on the Illusions of the Insane, translated by Mr. Liddell, reviewed 39
Etymology of the technical terms in physic . . . 67
Examinations at Apothecaries' Hall, method of reforming . 147
Examination at Apothecaries' Hall . . . . 465
Exposition of the False Medium, &c. reviewed . . 156
Face presentations; how to be treated . . . 154
Fistulæ in perineo, Mr. Brodie's method of treating . . 298
Fistulous openings between the bladder and vagina, method of treating 86
Fodéré on rape, quoted . . . . . . 368
Fœtal pulsation . . . . . . 276
Forceps, midwifery . . . . . 350
Fracture of the bones of the face, case of . . . . 233
Fractures of the pelvis, treatment of . . . . 231
French wines, Dr. Ayre erroneously censures . . 72
Fungated sores, J. Hunter on . . . . . 322
Gaubius; his table of doses . . . . . 119
George, Mr., his History of Small Pox, reviewed . . . 155
German tinder is made from the polyporus igniarius . . 104
Gold, internally administered . . . . . 120
Gonorrhœa in women; its treatment . . . . 85
and leucorrhœa, Dr. Davis's diagnosis of . . 89
Granville, Dr., his Graphic Illustrations of Abortion, reviewed 135 *et seq.*
Greenhow, Mr., his Description of an Apparatus, &c. reviewed . 153
Gunner with the silver mask . . . . . 199
Guthrie, Mr., his formulæ for ophthalmic ointments . . 10
on Inguinal and Femoral Hernia, reviewed . 126 *et seq.*
Gutta rosea, Alibert's observations on . . . . 146
Hahnemann, translation of the Organon of, reviewed . . 49 *et seq.*
Hatin, Dr., La Manœuvre de tous les accouchemens contre nature, &c. reviewed . . . . . . . 154
Hemlock and henbane, experiments on . . . . 215
Hemorrhage, J. Hunter's remarks on . . . . 317
Hernia in the old; the opening not affected by the contractions of the transversalis . . . . . . 131
Hernia radical, cure of, in Turkey . . . . 189
strangulated, Mr. B. Cooper's observations on . . 241
case of . . . . 242
Herpes of Alibert is equivalent to the psoriasis of Bateman . 145
Hip-joint, disease of the . . . . . . 234
remarkable case of . . . . 235
Hippocrates; quoted . . . . . . 319
Homœopathy; its fundamental axioms . . . . 49
Homœopathic cures, instances of . . . . . 51
treatment, success of the, shows the *vis medicatrix naturæ* 66
Hospital physicians and surgeons are pledged to the advancement of the medical art . . . . . . . . 105
Hottentot's bread . . . . . . . 460
Hull, the poor of, are very badly fed . . . . 71
Hunterian Reminiscences, edited by Mr. Parkinson, reviewed . 307
Hunter, J., the simplicity and beauty of his experiments . 311
Hydrophobia cured by the vapour-bath . . . . 433
Hypocondriasis, cases of, cured . . . . . 38
Hysteria, Dr. Davis's observations on . . . . 90
Hysterical retention of urine . . . . . 299
Illustrations of the Effects of Poisons, by Dr. Roupell, reviewed . 371
Infinitesimal doses . . . . . 63 *et seq.*
Infusional animalculæ have sometimes two hundred stomachs . 95

Inguinal hernia; its anatomy . . . . . 128
Inhalation of chlorine gas . . . . . . 188
Insanity; its sources . . . . . . 29
Insane illusions, cases of . . . . 40 *et seq.*
Insanity, who shall be the judge of . . . 347
Insane, the supposed, are confined too rapidly . . . 48
Intoxication, Dr. Ogston on . . . . . 335
Introductory Lecture, by Dr. Grant, reviewed . . . 373
Iodine, Mr. Twining on some of the effects of . . 328
Iritis, cases of . . . . . . . 14
syphilitic . . . . . . 15
Itch, insects causing the, microscopical characters of . . 218
Kennedy, Dr. Evory, on Obstetric Auscultation, reviewed . 272 *et seq.*
Key, Mr., on Strangulated Hernia, reviewed . . 139 *et seq.*
operates for strangulated hernia without dividing the sac *ib.*
Kreosote; its styptic properties . . . . . 439
Labium, abscess of . . . . . . 84
enlargement of . . . . . . 85
Laceration of the rectum; its causes . . . 106
intervertebral substance, case of . . 231
Langstaff's, Mr., case of medullary sarcoma . . . 291
Lawrence on Diseases of the Eye, reviewed . . 1 *et seq.*
Leucorrhœa, Dr. Jewel's remarks on . . . 332
in young children taken for gonorrhœa . . 369
Licentiate, physicians, petition of . . . 221
Lichens rarely grow in dark places . . . . 102
their geographical distribution . . . *ib.*
Life, J. Hunter's account of . . . . . . 309
Ligature of the carotid arteries in epilepsy . . . 328
of the common carotid artery, cases of, by Mr. Mayo . 410
Lithotomy, Mr. Brodie's observations on . . . 303
by the high operation, case of, by Mr. Key . . 388
cases of, successfully performed by Mr. Keith . 338
Mr. Liston . . 339
Lloyd, Mr., his case of jaundice with fatty discharge from the bowels 286
Locked jaw, J. Hunter on . . . . . 314
Logic necessary to medical practitioners . . . 313
London Med. and Phys. Journal; its judicious opinions of Dr. Davis's style 83
Medical Society; account of its proceedings . . 468
Practice of Midwifery, edited by Dr. Jewel, reviewed 331 *et seq.*
Longevity of authors . . . . . . 219
Lonsdale, Mr., his apparatus for fractured lower jaw . . 234
Lunatic asylums, cruelty in . . . . . 34
Macilwain, Mr., on Porrigo, reviewed . . . 293 *et seq.*
Madness, difficulty of defining . . . . . 26
Magendie, Précis Elémentaire de Physiologie, reviewed . 245 *et seq.*
Magnitude, notion of, now acquired . . . 250
Malaria the cause of the cholera . . . . . 69
Marchantia polymorpha, Mr. Burnett's account of . . 216
Mayo, Mr., on Diseases of the Rectum, reviewed . . 105 *et seq.*
on strangulated hernia . . . . . 379
on ligature of the common carotid . . . 410
Medical books may be divided into three classes . . 229
Medico-Chirurgical Transactions, Vol. XVII., Part I., reviewed 278 *et seq.*
Medullary sarcoma, case of . . . . 291
Melitagra; cured by ioduro-sulphureous lotions . . 146
Memory; its return . . . . . . 30
Menstruation, case of late . . . . . 87
genuine cannot occur during pregnancy . . 88
Mercantile men; their habits unwholesome . . . 210
Meteorological Register . . . . 228 and 472
Midwifery has always been liberally fostered . . . 349
Miller's Gardener's Dictionary, reviewed . . . 375
Milligan, Dr.; biography of . . . . . 471

Mistakes as to the presentation in labour . . . 188
Murray, Mr.; his Manual of Chemical Experiments, reviewed 151 *et seq.*
Musci, geological distribution of the . . . . 209
Muscular sense, observations on the . . . 247
Nascatapoor, or Nose-cut-town . . . . 265
Nature cures homœopathically . . . . 58
Neck of the femur; case of its fracture within the capsule at an early age 292
Nervous fluid, Mr. Earle's theory of the . . . 125
Nerves of sense, impression made on, may be general stimuli . 251
Nitrate of ammonia, composition of . . . 369
of silver; its effects in blackening the skin . . 122
Nose, on the removal of morbid enlargements of the integuments of the, by Mr. Dalrymple . . . . . 395
Notices . . . . . 228 and 472
Nouveau Formulaire des Hôpitaux par M. M. Milne Edwards et P. Vavasseur, reviewed . . . . 118 *et seq.*
Obituary . . . . . .228 and 471
Obturator made of different metals, effects of an . . 436
Oculists; their superiority over hospital surgeons in operating on the eye 3
Œsophagotomy performed by Mr. Arnott . . . 290
Oily or fatty stools, cases of . . . . 279 *et seq.*
Ophthalmia, treatment of, when the acute stage is passing into the chronic 7
chronic; its treatment by astringents . . 8
Egyptian; its history and statistics . . . 12
gonorrhœal, Mr. Lawrence's over-vigorous treatment of 13
surgery; its history . . . . 3
Opium, medicines incompatible with . . . 120
Order of the eruption of the first teeth . . . 453
Outlines of Botany, the plan of, original and good . . 93
Ovarian dropsy, cases of . . . . 157 and 158
Parturition, cases of easy, in deformed women . . 192
easy among savages . . . . 461
rapid; as connected with forensic medicine . 462
Peritonitis, with fœcal abscess, case of, by Dr. Burne . . 385
Phthisis, case of, by Dr. Stroud . . . . 401
Physiology of the growth and reparation of bone . . . 229
Piles, Mr. Mayo's account of . . . . 109
method of removing by the ligature . . . 112
remarkable case of, outward . . . . 113
Plants, list of exotic, growing in the open air at Torquay . . 338
Pope; his constitution . . . . . 205
Post-mortem examination of a painter . . . 441
Practice of physic should be open to every one . . 373
Pregnancy, doubtful case of . . . . . 277
Preparation of animals for collections . . . 207
Prescriptions in Gölis on hydrocephalus . . . 193
Preston, Mr., on ligature of the carotids in epilepsy . . 328
Principles and Illustrations of Morbid Anatomy, by Dr. Hope, reviewed 372
Prognosis, Dr. Uwins objects to the word . . . 31
Prometheus, a distiller . . . . 449
Prolapus ani, Mr. Mayo's account of . . . . 109
Pruritus pudendi to be treated with solution of nitrate of silver . 86
Prussian blue and saccharine matter contained in morbid urine . 211
Psora used by Hahnemann for chronic disease in general . 62
Puerperal fever to be distinguished from peritonitis . . 334
insanity commonly cured . . . 32
mania described by Dr. Jewel . . . 334
Quadrupeds, on the walk of . . . . 195
Quetelet on the weight of man at different ages . . . 204
Quain's, Dr., Anatomical Plates, reviewed . . . 374
Rape, Dr. Beattie on . . . . . 367
is rarely possible . . . . . 368
Rayer on Diseases of the Skin, reviewed . . 121 *et seq.*
his classification of cutaneous diseases . . 121

Rectum, rupture of the, into the vagina, cases of . . . . 106
Recto-vaginal fissure cured by an operation . . . . 107
Rennie's, Professor, Alphabet of Scientific Chemistry, reviewed 256 *et seq.*
Report of the obstetric practice of the Wellesley female institution, by Dr. Maunsell . . . . . . . 336
Restoration of the nose . . . . . . 267 *et alibi.*
Retinitis, diagnosis of . . . . . . 15
Rice, Dr. Tytler on, reviewed . . . . . . 371
bad, the supposed cause of Asiatic cholera . . . . 436
Ringworm treated by Mr. Wigan with pyroligneous acid . . 437
Roberts, Dr. C. J.; his case of softening of the brain . . 166
Sand in the Urine, Mr. Brodie's observations on . . . 300
Sangrado rivived in modern hospitals . . . . . 7
Salmon, Mr., on Stricture of the Rectum, reviewed . 149 *et seq.*
Scotch ascendancy, the proposed, in the college of physicians . 464
Sensibility and touch, Sir C. Bell's remarks on . . . 361
Seton dangerous in bronchocele . . . . . 24
Signs of pregnancy and delivery, Dr. Montgomery on . . . 366
Silent patient frightened into talking by galvanism . . 39
Sloth, Sir. C. Bell's observations on the . . . . 359
Snails still sold for the consumptive . . . . . 194
Soldier's meals, formerly, inspected . . . *ib.*
Soufflet, the placental . . . . . . 374
Stanley, Mr., his case of fracture of the neck of the femur within the capsule at an early age . . . . . . 292
Statistics of European mortality . . . . . 448
Steel busks . . . . . . . 442
Strangulated hernia, heads of, clinical lectures on, by Mr. Mayo . 379
Stricture, imaginary, made real by the bougie and sound . . 163
permanent, situation of . . . . 161
Stroud, Dr., his case of empyema . . . . . 174
St. Vitus's dance . . . . . . 449
Suicide prevented by exposing the bodies of the dead . . 37
Sulphate of cadmium; its use in diseases of the eye . . . 428
Supernumerary cervical ribs, case of, by Dr. Dymock . . 337
Sus Ethiopicus; adaptation of its frame to its mode of life . . 363
Swan's, Mr., New Method of making Anatomical Preparations, reviewed 355
Syllabus of a Course of Lectures on the Principles and Practice of Surgery, by Frederick Tyrrell, Esq., reviewed . . . . . 370
Sympathetic, the great, Magendie's observations on . . . 254
Tea, an index of the diffusion of refinement . . . . 29
Thoughts on Medical Reform, reviewed . . . . . 373
Thyröid artery tied for the cure of bronchocele . . . 18
Torquay, account of its climate, by Dr. Coldstream . . . 337
Transactions of the Med. and Phys. Society of Calcutta. Vol. vi. reviewed 323
Traumatic tetanus, case of . . . . . . 192
case of, cured by Dr. Murray, by dividing the posterior tibial nerve . . . . . . . 328
Tyrrell, Mr., on permanent strictures of the urethra . 160 and 414
Uterus, rupture of the, Dr. Campbell's observation on . . 352
Uwins, Dr., on Insanity, reviewed . . . . 25 *et seq.*
Van Helmont, quoted . . . . . 308 *note.*
Venesection not to be practised on the poor of London . . 7
rarely required in hysteria . . . . . 92
Vision, the theory of . . . . . . 248
Vital power, transference of . . . . . . 441
principle, not admitted by Magendie . . . . 245
Vinum opii, why the College of Physicians diminished its strength . 11
Want of pulse in the right arm . . . . . 189
Weight of man at different ages . . . . . 204
Worms found in the blood of a boy . . . . 364
Young, Dr., his ingenious formula for proportioning doses . . 119
Dr. Thomas, his unrivalled acquirements . . . 459
Zoological mistake of President Jefferson . . . . 360

